# Mapping HIV/STI behavioural surveillance in Europe

**DOI:** 10.1186/1471-2334-10-290

**Published:** 2010-10-04

**Authors:** Françoise Dubois-Arber, André Jeannin, Brenda Spencer, Jean-Pierre Gervasoni, Bertrand Graz, Jonathan Elford, Vivian Hope, France Lert, Helen Ward, Mary Haour-Knipe, Nicola Low, Marita van de Laar

**Affiliations:** 1 Institute of Social and Preventive Medicine (IUMSP), University Hospital Center and University of Lausanne, Lausanne, Switzerland; 2 City University, London, UK; 3 London School of Hygiene and Tropical Medicine, London, UK; 4 Institut national de la santé et de la recherche médicale, Villejuif, France; 5 Imperial College, London, UK; 6 Freelance Consultant, Geneva, Switzerland; 7 Institute for Social and Preventive Medicine, Bern, Switzerland; 8 European Centre for Disease Prevention and Control, Stockholm, Sweden

## Abstract

**Background:**

Used in conjunction with biological surveillance, behavioural surveillance provides data allowing for a more precise definition of HIV/STI prevention strategies. In 2008, mapping of behavioural surveillance in EU/EFTA countries was performed on behalf of the European Centre for Disease prevention and Control.

**Method:**

Nine questionnaires were sent to all 31 member States and EEE/EFTA countries requesting data on the overall behavioural and second generation surveillance system and on surveillance in the general population, youth, men having sex with men (MSM), injecting drug users (IDU), sex workers (SW), migrants, people living with HIV/AIDS (PLWHA), and sexually transmitted infection (STI) clinics patients. Requested data included information on system organisation (e.g. sustainability, funding, institutionalisation), topics covered in surveys and main indicators.

**Results:**

Twenty-eight of the 31 countries contacted supplied data. Sixteen countries reported an established behavioural surveillance system, and 13 a second generation surveillance system (combination of biological surveillance of HIV/AIDS and STI with behavioural surveillance). There were wide differences as regards the year of survey initiation, number of populations surveyed, data collection methods used, organisation of surveillance and coordination with biological surveillance. The populations most regularly surveyed are the general population, youth, MSM and IDU. SW, patients of STI clinics and PLWHA are surveyed less regularly and in only a small number of countries, and few countries have undertaken behavioural surveys among migrant or ethnic minorities populations. In many cases, the identification of populations with risk behaviour and the selection of populations to be included in a BS system have not been formally conducted, or are incomplete. Topics most frequently covered are similar across countries, although many different indicators are used. In most countries, sustainability of surveillance systems is not assured.

**Conclusion:**

Although many European countries have established behavioural surveillance systems, there is little harmonisation as regards the methods and indicators adopted. The main challenge now faced is to build and maintain organised and functional behavioural and second generation surveillance systems across Europe, to increase collaboration, to promote robust, sustainable and cost-effective data collection methods, and to harmonise indicators.

## Background

The epidemics attributable to the Human Immunodeficiency Virus (HIV) and to other sexually transmitted infections (STI) remain a significant public health problem in Europe. The incidence of HIV infections is not decreasing, nor is it even stabilized in many populations, such as men who have sex with men (MSM), injecting drug users (IDU), or certain migrant populations, such as persons from Sub-Saharan Africa [1,2]; furthermore, the reported incidence of several STI is increasing [3-8].

Since the beginning of the HIV/AIDS epidemic, the importance of monitoring risk behaviour in various target populations has been recognized and different indicators have been proposed [9]. In the late 1980's, several European countries began to collect indicators of behaviour in various populations exposed to risk [10-13], such as MSM [14-17], IDU [18-20], the general population [21,22], youth [23] and sex workers (SW) [24,25]; the concept of behavioural surveillance (BS) progressively emerged [26,27].

The utility of such behavioural surveillance data in guiding programme development, especially in conjunction with biological surveillance data, became apparent during the 1990's. The Joint United Nations Programme on HIV/AIDS (UNAIDS)/World Health Organization (WHO) has defined second generation surveillance (SGS) [28,29] as the «regular, systematic collection, analysis and interpretation of information for use in tracking and describing changes in the HIV/AIDS epidemic over time». This requires procedures to ensure a combined analysis of both behavioural surveillance (BS) data and data obtained from biological surveillance of HIV/AIDS and STI. BS allows for the monitoring of risks related to transmission of HIV and STI at the population level and provides a key source of information not only to understand the drivers of epidemics, but also for advocacy and for the planning and evaluation of prevention interventions [30,31]. The type of BS to be conducted, in particular the populations to be included in the surveillance, depends of the type of HIV epidemic (generalised, concentrated, low level) [32]. Most European countries have a concentrated type of epidemic but some have a low-level type.

At the global level, behavioural indicators in various populations are included in the set of indicators defined to monitor commitment to the United Nations General Assembly Special Session on AIDS (UNGASS indicators) [33]. At the European level, the European Monitoring Centre for Drugs and Drug Addiction (EMCDDA) has proposed a set of core behavioural indicators for use with IDU[34].

The formalisation and organisation of BS in Europe is in progress [31,35], but without any review of current practices having been conducted. There has been little consideration of behavioural surveillance systems in the scientific literature [36] and discussion on BS has mainly appeared only in journal supplements [37-40]. In 2008, the European Centre for Disease Prevention and Control (ECDC) therefore commissioned an international team of experts to produce an in-depth analysis of BS related to HIV and STI in Europe and to develop a framework for the implementation of a key set of behavioural indicators. The specific objectives of the study were to provide an inventory of existing national BS systems and their functioning in Europe, including identification of the populations included, and the data collection methods and indicators used in each system. This article presents the resultant mapping of BS in Europe, based on a survey of EU/EFTA countries [41]. Surveillance is examined at the system level and in the following populations: general population, youth, IDU, MSM, persons living with HIV/AIDS (PLWHA), SW, STI clinic clients, migrants and ethnic minorities.

## Methods

An international team of researchers was established with expertise in behavioural surveys in each of the 8 designated populations. The survey of all EU/EFTA countries was conducted from June to October 2008. Information was gathered on: the overall surveillance system, the specific populations surveyed and the topics covered and main indicators in use for each population. The data collection instrument consisted of 9 separate questionnaires sent to one identified contact person in each country. One questionnaire concerned the national behavioural and second generation surveillance system(s) as a whole, and there were an additional eight questionnaires covering each of the eight specific population groups. The questionnaires, provided in word format using dropdown lists, included both open and closed questions (see Additional files 1 and 2). In the system-related questionnaire, information was requested on:

- the existence and definition of a national or regional BS system(s); indicating which populations were included and the national key behavioural indicators;

- where in place, the functioning of the second generation system at national or regional level, including level of formalisation and documentation, interlinking of systems, responsibility, financing, analysis, dissemination and use of results, and sustainability.

In the questionnaires on specific populations, it was first asked whether a surveillance system was in place for this population and information was then requested on the existence of behavioural surveys (or other types of data collection used for surveillance) conducted since 1985. For each relevant survey (or other type of data collection) indicated, information was requested on:

- methodology used, including year, sampling method, data collection method, and main topics covered;

- main indicators followed;

- related publications.

### Procedure and analysis

A letter announcing the survey and inviting countries to participate was sent from the ECDC director to the directors of the competent bodies for surveillance in the Member States and to the national ECDC contact points. The set of 9 questionnaires was then sent by email to the contact persons responsible for HIV surveillance (as nominated by the ECDC competent bodies for surveillance) in all EU Member States and European Free Trade Association (EFTA) countries (n = 31). Individual questionnaires could be passed on to the relevant national specialists for completion; the responsible contact person then collected and sent back all questionnaires.

The returned questionnaires and accompanying material were included in a secure online database accessible only to the international project team. The analysis for each specific population was conducted according to a commonly agreed framework by the relevant team member. The team had regular telephone conferences during the analysis.

In February 2009, a first draft of the mapping was presented and discussed at a Behavioural Surveillance Expert Meeting. Fifty participants, including experts in behavioural surveys in the various populations and experts from other international organisations (EMCDDA, WHO, UNAIDS), reviewed and gave advice on the methods and indicators proposed for each population. A revised draft of the mapping was then sent for validation to the persons who had initially collated the questionnaires. Eleven countries then provided complementary information.

The study collected scientific and administrative meta-data on the organisation of national behavioural and second generation surveillance systems, and on the surveys undertaken in this context. As no data was collected on individuals, review by an ethics committee was not necessary. This study was conducted on behalf of, and with the approval of the European Centre for Disease Prevention and Control.

## Results

Twenty-eight of the 31 countries surveyed (90%) returned the questionnaire set; the remaining 3 countries did not respond within the prescribed deadline (Bulgaria, Romania, and Portugal; Bulgaria later sent a summary of its surveillance system in place since 2004, but these data could not be included in the current analysis). Most questionnaires were fully completed.

### Coverage of behavioural surveillance

**Figure 1:**
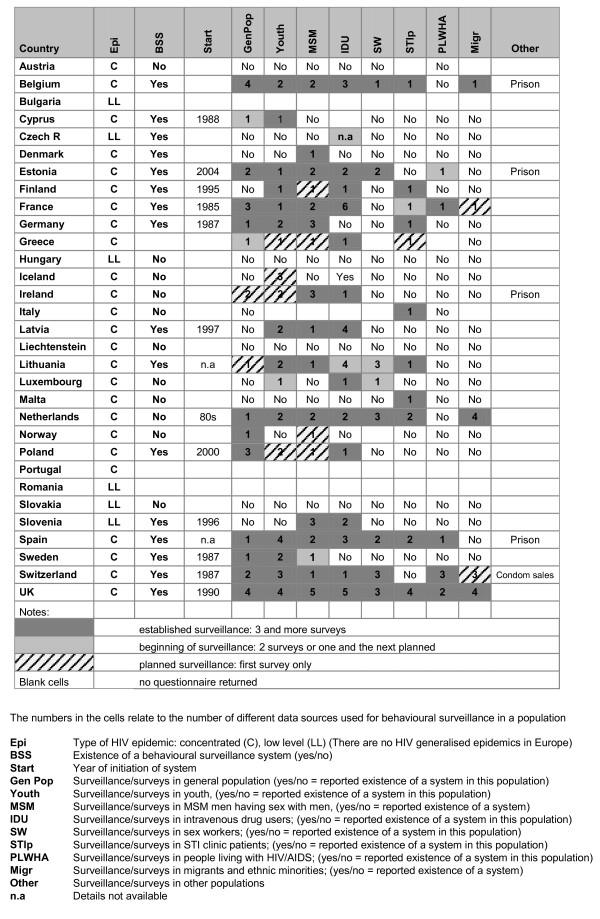
Behavioural surveillance systems in EU/EFTA countries: mapping of HIV/STI behavioural surveillance in Europe, 2008

Figure 1 gives an overview of existing systems in Europe, across different countries and populations, with a summary description of the HIV/STI BS system in each country. As used here, the term surveillance refers to data collection through several consecutive surveys. This may be organised surveillance through repeated surveys or less organised «systems» based on consecutive data collections in the same type of population. In Figure 1, the intensity of the colour in each cell refers to the degree of establishment of the system and the number refers to the number of different data collection methods/sources included in the system for a given population.

Fifteen countries report having an established HIV/STI BS system (Belgium, Cyprus, Denmark, Estonia, Finland, France, Germany, Latvia (only for IDU), Lithuania, Poland, Slovenia, Spain, Sweden, Switzerland, United Kingdom), and one additional country (the Netherlands) reports established BS in several specific populations, but no formal system. The remaining 12 countries report no BS system. However, in most of these countries, collection of behavioural data is nonetheless reported for one or more populations.

There are wide differences among countries as regards the year of initiation of BS: this extends from 1985 (France: MSM) to the early 2000's (Poland 2000, Estonia 2004: several populations).

The populations most surveyed are the general population (13 countries reporting a system [=Sy]; 4 reporting none, but nonetheless conducting surveys or collecting other type of data [=sv]), youth (Sy: 13; sv: 5), MSM (Sy: 14; sv: 4) and IDU (Sy: 17; sv: 0). Where a system is in existence, MSM and IDU are the most regularly surveyed populations. Sex workers (Sy: 6; sv: 2), patients of STI clinics (Sy: 9; sv: 1) and PLWHA (9 countries report a system, but in 3 of these, no data are collected on risk behaviour) are less surveyed, and few countries report having undertaken BS among migrant or ethnic minority populations (3 countries with a reported system; 2 with planned BS).

In most countries and populations, BS relies on one or two data collection sources per population. In some countries, however, more sources are used. For example, the UK reports relying on five ongoing surveillance surveys among MSM. When a population is difficult to reach, a diversity of sources may be used. Hence, in Switzerland, specific questions on «paying for sex» or «being paid for sex» are included in surveys among the general population, MSM and IDU. Similarly, a diversity of recruitment sites and populations surveyed is adopted in BS among migrants in the Netherlands. Spain obtains data on youth through surveying a diversity of age groups and collecting data from surveys with different focal points, such as sexuality, general health, or drugs.

### Completeness of Systems

Since the start of the HIV/AIDS epidemic, several Western European countries with early concentrated epidemics have conducted behavioural surveys in order to identify risk behaviour and monitor progress in prevention. These have taken place in a number of different populations, essentially in the general population, youth, MSM and IDU. In some countries or regions, these surveys were undertaken and repeated with the intention of constituting a coherent BS system. Switzerland and Catalonia in Spain have such long-term integrated systems. In other countries, the «system» was constructed progressively, with the addition of new surveyed populations over time. Although the surveys were sometimes analysed together as a periodic review exercise, there was not always a clear surveillance objective. France, Germany, the Netherlands and the United Kingdom generally have such established surveillance in four or more populations, but this activity is not coordinated into a system.

In Eastern European countries with recent concentrated epidemics, mainly in IDU (such as Estonia, Lithuania, Latvia), formal comprehensive systems have been set up recently, using from the outset the concept of second generation surveillance. This has often involved the collaboration and funding of international agencies. These systems mainly include small-scale surveys in 2-4 populations.

In the other countries (Belgium, Cyprus, Denmark, Finland, Latvia, Slovenia, Spain and Sweden), systems were established during the 1990 s, and include national and regional/local surveillance and/or a small number of populations.

Even in countries with formalised or long established BS, there is often no established regularity in the timing of BS in the different populations, possibly indicating instability of the system. For example, in some Western European countries, surveys in the general population and MSM were conducted more frequently at the beginning of the AIDS epidemic than during recent years. Consequently, the term «system» does not necessarily imply data collection at regular intervals.

### Organisation and functioning of BS

The degree of formalisation of BS within a system is very unequal across the different countries (Table 1). Among the 16 countries reporting or having *de facto *BS, only 8 declared the existence of a document describing this system or formalising its existence: Cyprus, Estonia, Latvia, Lithuania, Poland, Switzerland and United Kingdom. Most of these countries have recent, concentrated HIV epidemics. The system is generally mentioned in the national strategic HIV/AIDS plan or in the Monitoring & Evaluation Plan (M&E plan).

**Table 1:** Formalisation and organisation of behavioural and second generation systems in countries reporting behavioural surveillance (n = 16).

	Behavioural surveillance system		Second generation system		
	
	Formalisation in documents	Organisation	Existing	Formal	Management/coordination body and functioning
**Belgium**	No	Informal, mix of national and regional repeated or small scale one/off studies	No	-	-

**Cyprus**	HIV/AIDS strategic plan	No management/coordination body	Yes, partly	Yes	Information from BS and biological systems is analysed and interpreted informally by program officials. A recent report on period 1986-2007 has been produced

**Denmark**	No	Surveillance only in MSM	No		

**Estonia**	M&E system, national AIDS strategic plan	Coordination by a government agency	Yes	Yes	Separate bodies for behavioural and biological surveillance, coordination by the National Institute for Health Development Agency with centralised production and dissemination of reports

**Finland**	No	Under the responsibility of a government agency	No	-	Under the responsibility of the National Public Health Institute

**France**	No	No centralized body: various national agencies (surveillance, research, prevention) and other institutions are involved inbehavioural surveillance.	Yes	No	Coordination by the Institut de Veille Sanitaire (InVS). Reports or syntheses of studies are issued periodically

**Germany**	No	Informal coordination between the Federal Ministry of Health, a health agency and research institutions	Yes, partly	No	Organised informally by a network of institutions

**Latvia**	Regulation for registration of infectious diseases	Under the responsibility of a government agency	Yes, for IDU only	Yes	Under the responsibility of the Public Health Agency

**Lithuania**	National AIDS programme and Resolution on STI surveillance	Under the responsibility of the Ministry of Health (MOH)	Yes, partly	Yes	Management by the MOH

**Netherlands**	No	No centralised body: cooperation between the National Institute for Public Health and the Environment and the HIV/STIs Agency	Yes, partly	Yes	Cooperation within a network of institutions; reports are issued regularly

**Poland**	National programs on AIDS and on addictions	No centralized coordination, collaboration between agencies	Yes partly	Yes	Information comes from different sources, and is sometimes, but not routinely, used to interpret the epidemiological situation. Reports are published on the government site

**Slovenia**	Institute of Public Health internal protocols	Under the responsibility of the Institute of Public Health of the Republic of Slovenia	Yes	Yes	Institute of Public Health is responsible for the analysis, interpretation and dissemination of SGS information to many audiences (MOH, media, professionals, population)

**Spain**	HIV multisectoral plan	Under the responsibility of the Secretariat of the National Plan on AIDS, Ministry of Health	Yes	Yes	The Secretariat of the National Plan on AIDS collects and publishes available information

**Sweden**	No	Under the responsibility of a government agency of the National Board for Health and Welfare	Yes, for general population	-	Collaboration within a network of institutions; summary reports are produced by the National Board of Health and Welfare for the government, and then sent to all health authorities in the country

**Switzerland**	National AIDS program, protocols	Under the responsibility of the University Institute of Social and Preventive Medicine and financed by the Federal Office of Public Health	Yes	Yes	Coordination is assumed by the Federal Office of Public Health (FOPH). All information pertaining to SGS is presented and discussed in a special working group-including FOPH, non-governmental organisations, partners from biological and behavioural surveillance. Reports are issued periodically

**UK**	Scientific article	No centralized body. Data produced by a network of agencies.	Yes, partly	Yes	No central coordination and dissemination of data; this is left to individual institutions and/or research groups to synthesise and publish

Mapping of HIV/STI behavioural surveillance in Europe, 2008.

The organisation of BS also shows diversity. In countries with formalised BS, its organisation and coordination is based in the Ministry of Health (MOH) (e.g. Lithuania), or in a National Health Agency (e.g. Estonia), or, in one case, in a university (Switzerland). In countries with less formalised systems, coordination occurs through a network of institutions: government agencies, universities, NGO's (e.g. United Kingdom, France).

In Western Europe, the government is generally the main source of funding for BS. In certain cases, additional resources come from research. A few countries cite international agencies, such as the Global Fund on AIDS, Tuberculosis and Malaria (GFATM), as partners in the funding of BS.

Twelve of the sixteen countries with established BS report that sustainability is assured or probably assured. Sustainability and cost are the main problems mentioned by countries regarding BS.

### Second generation surveillance (SGS)

Among the 16 countries reporting BS, 13 also report the existence of SGS. Two additional countries report SGS in one population: Latvia (in the IDU population) and Sweden (in the general population).

Six countries (Estonia, France, Lithuania, Slovenia, Spain, and Switzerland) report a formalised BS system, with a management or coordinating body, organised in one of two different ways:

- management by MOH or by a governmental agency, with collection and synthesis of information from diverse sources and production and dissemination of reports;

- coordination by MOH of discussion of SGS results by a group of diverse stakeholders, including agencies/universities producing the data, MOH representatives (epidemiology, prevention, evaluation) and AIDS NGO's.

Five countries report having a SGS system that is not formally organised: in Germany, the Netherlands and the United Kingdom, SGS is carried out by a network of institutions without centralised coordination. Reports are produced regularly (The Netherlands); their production and dissemination may be left to the individual institutions (United Kingdom).

In countries reporting SGS, data seem to be used for a variety of purposes, with different audiences in mind. The three most frequent uses are: interpreting trends in HIV incidence and prevalence, identifying the drivers of the epidemic and measuring indicators of progress in programme development. SGS is less used in programme planning, in projecting future interventions or as an advocacy tool to increase resources and expand response.

### Methods used for behavioural surveillance in the different populations

Table 2 shows the methods used for BS in the different populations. For the general population and youth, surveillance is based on surveys in a representative sample of the population. A mix of data collection methods/sources was in use in several countries (e.g. comprehensive sexuality surveys conducted at long intervals complemented by more regularly conducted KABP). Questions on sexuality included in general health surveys are the main surveillance mode in use for young people, this population being either specifically surveyed or included as a subsample of the general population.

**Table 2:** Methods used in behavioural surveillance: number of countries using as main method(s).

	General Population	Youth	MSM	IDU	SW	STI patients	PLWHA	Migrants
**Representative designs**								

Sexuality module in general health surveys	6	14			2			2

Specific KABP survey	8	10						

Comprehensive sexuality/reproductive health surveys	8	6						1

Addiction focused surveys	2	5						

Surveys using respondent driven sampling for hard-to-reach populations			2	2	1			

**Non representative designs**								

Service based data collection				11	5	9	3	1

Internet surveys		1	14				2	

Venue-based/community based surveys			12	8	4		3	5

Cohort studies							2	

Mapping of HIV/STI behavioural surveillance in Europe, 2008.*

* More than one method may be used for surveillance in a population

There is a long tradition of MSM surveillance in Europe. Internet surveys are progressively replacing or complementing venue-based surveys; respondent driven sampling (RDS) has recently been used in countries where MSM are still difficult to reach (Estonia, Greece).

BS among IDU is focused on the collection of data from those in contact with services, either through routine collection of assessment data on those entering treatment or through cross-sectional surveys of those in contact with a range of health or social services, such as needle exchanges and drop-in centres. A small number of community-based surveys have been conducted using a range of methods.

In sex workers, surveillance is mainly service-or venue-based. Several countries also use data collected in other populations, such as MSM or IDU, and two countries use data on sex worker clients collected in general population surveys.

In PLWHA, services and venues are also the main recruitment sites, and surveys in specific populations (IDU and MSM) are also used for surveillance of PLWHA in these populations. Cohorts are used for BS in 2 countries.

For migrants, where established surveillance is rare, general population surveys with questions on nationality or country of origin, allowing for identification of migrants, and/or community-based surveys are the main sources of data. The mapping exercice also revealed the extent to which different terms and concepts are used to refer to «migrants».

For STI clinic patients, surveillance is, by definition, service-based and relies on either routinely collected data or point prevalence surveys.

### Indicators

Analysis of topics covered in the various surveys showed much convergence across countries and across populations on the most important indicators, even if the degree of formulation is still uneven. Harmonisation has taken place in certain populations (in IDU with EMCDDA indicators, in MSM through different initiatives [42]) and, more generally, through the UNGASS reporting process. However, there is still enormous variation between countries in the specific indicators used with regard to content and wording, reference period used, and so on. To illustrate, in young people, 11 different indicators of condom use were identified, including condom use at last sexual intercourse, condom use at last intercourse among those who had more than one partner in the last 12 months, condom use with steady partner, condom use in spontaneous sexual contact, condom use in new relationships. In order to further the harmonisation process, five core indicators have been proposed that are common to all populations. These are coherent with existing international recommendations and cover:

- the number of sexual partners in the last 12 months

- condom use at last intercourse (in the last 12 months) with the identification-in a consecutive question-of the type of partner with whom the last intercourse took place (stable, casual, or paid)

- experience of HIV testing, constructed on the basis of three questions (ever tested, date and result of the last test)

- sex work: having paid for sex in the last 12 months and use of condom at last paid sex

- a composite indicator of knowledge (UNGASS indicator number 13).

A common set of socio-demographic indicators are also proposed: level of education, nationality or ethnic origin and sexual orientation.

## Discussion

Europe is rich in expertise and experience in behavioural studies related to HIV and STI, especially among MSM, IDU, the general population and youth. In many countries, there has been a progressive accumulation of surveys or other types of data collection constituting, *de facto*, the necessary elements to build a BS system. However, even when the material is available, the concerted organisation of this into a formal, comprehensive and sustainable system has only taken place in a limited number of cases. This is particularly true as regards second generation systems, a significant part of which have only a low level of fomalisation and a moderate level of dissemination and use. This is reflected in the paucity of published articles and of available reports on BS systems; data on behaviour in specific populations being more frequently the object of publications.

Movement towards simplification of data collection is noticeable in various populations, as exemplified by the increased use of internet-based surveys in MSM, service-based data collection in IDU, and in the use of general health surveys to collect data on sexual behaviour and drug use in the general population and in youth. These newer approaches offer advantages in terms of sustainability and cost provided that comparison over time can be maintained.

In MSM and IDU, where there is the longest experience of BS, the length and robustness of trends and the relatively low cost of non-probablistic methods of sampling (venue-, internet- or service-based) can explain the paucity of experience with RDS and the probable reluctance to adopt this recently promoted method for BS in these populations [43]. Favouring less costly data collection methods over RDS would seem a reasonable choice in the case of mature epidemics.

It is not easy to evaluate the adequacy of BS in European countries: in many cases the identification of populations with risk behaviour and the selection of populations to be included in a BS system has not been formally defined, or is incomplete. Several gaps may nevertheless be identified:

• Some countries still have no data on MSM or IDU. Very few countries have collected data on sex workers and migrants, two populations difficult to reach and/or to identify reliably, and practically none has BS installed on a long-term basis. Few BS systems are in place in populations that are clearly identified as potentially at-risk of acquiring or transmitting infections and accessible through the health system, such as STI patients and PLWHA.

• In several countries, the formalisation and functioning of the system and its use are less developed than might be expected, given the opportunities.

There are many challenges regarding the future of BS in Europe:

One of the challenges in the years to come will be to reinforce or maintain BS. Europe, particularly Western Europe, faces a decrease in the level of priority accorded to HIV (for example, in reaction to potential new epidemics, such as AH1N1), with a consequent decrease in the availability of funding for HIV prevention and research, including behavioural surveys. In certain Eastern European countries, international funding provided to initiate surveillance is decreasing. The necessity of maintaining BS may be challenged. Yet, there are many reasons to maintain BS in the Europe:

- As mentioned above, the epidemiological situation regarding HIV and other STI is worsening in many countries. In addition, there is a continuous increase in the pool of HIV infected people.

- A decrease in the use of protection and the development of alternative HIV/STI-related patterns of behaviour (risk reduction, stopping condom use under certain treatment conditions [44]) has begun to appear and deserves close monitoring.

- There are concerns regarding access to prevention and care, especially for marginalised populations, such as certain categories of migrants [45] and IDU [46]; BS could be used for planning interventions and advocating for resources.

- The sustainability of existing systems is not always guaranteed.

The cost of BS is one element threatening sustainability. Attempts have been made to reduce costs, while maintaining the availability of valid and reliable behavioural trends, and may serve as examples. These include: use of internet (MSM) and service-based surveillance (wherever possible); increased collaboration with existing PLWHA cohorts; insertion of sexuality modules in general health surveys replacing KAPB surveys; use of proxy data, such as information on SW obtained from clients in general population surveys.

The frequency of data collection should also be reconsidered. In several populations, such as the general population and youth, intervals between data collection may extend to 4-6 years, while in populations with higher HIV incidence, shorter intervals and more flexibility are needed. Qualitative studies may also be used to provide information on developments in specific populations and to facilitate decisions regarding the necessity of conducting surveys.

Our study has some limitations. The information delivered by the questionnaire was self-reported and the responses varied greatly as regards the level of detail included in the description of systems. The person to whom the questionnaire set was sent in each country was the ECDC contact person at national level for HIV biological surveillance; there is currently no specific ECDC contact person for behavioural surveillance. This person may have been unaware of the existence of surveys in some or all populations of interest, whether organised or not in a behavioural surveillance system, and may have given inadequate answers or may not have passed the population-specific questionnaires to the relevant national specialists. This would result in underreporting of existing surveys. However, it is very unlikely that any organised national behavioural surveillance would be unknown to the ECDC contact person. Furthermore, this survey was launched with a letter from the ECDC director, thus indicating a high level of importance. The report was also circulated to countries for validation, offering a further occasion for verification.

Few original questionnaires used for surveillance were sent by the countries. Nevertheless, most countries sent useful complementary information, such as references to documents or publications describing their systems.

## Conclusions

The main challenge now faced is to build and maintain organised and functional behavioural and second generation surveillance systems at national level, with the following characteristics. First, there should be a regular assessment of the appropriateness and quality of the system. Second, a designated body should be set up, with clear responsibility for data coordination, collection, analysis and dissemination. This body should be qualified to make decisions regarding the simplification or extension of the system, on the basis of epidemiological and behavioural trends, cost, sustainability, quality and robustness of the system.

## Competing interests

The authors declare that they have no competing interests.

## Authors' contributions

FDA: team leader, direction of the research, responsible for the analysis of data on systems and on youth, writing of the article. AJ organised the survey and prepared the questionnaire

BS participated in the preparation of the questionnaire and was responsible for the analysis of data on general population. JPG participated in the preparation of the questionnaire and organised the technical workshops. BG conducted the literature review and participated in the analysis of data on youth. JE was responsible for the analysis of data on men having sex with men. VH was responsible for the analysis of data on injecting drug users. FL was responsible for the analysis of data on people living with HIV. HW was responsible for the analysis of data on sex workers. MHK was responsible for the analysis of data on migrants. NL was responsible for the analysis of data on STI clinics' clients

MvdL coordinated the work at ECDC with Member states and participated in the set up of the survey. All authors reviewed the article and read and approved the final manuscript.

## Pre-publication history

The pre-publication history for this paper can be accessed here:

http://www.biomedcentral.com/1471-2334/10/290/prepub

## Supplementary Material

Additional file 1 **questionnaire related to behavioural surveillance in the general population**. This questionnaire collects information on the existence and the characteristics of behavioural surveillance conducted in the general population in a given country. Click here for file

Additional file 2 **questionnaire related to the behavioural surveillance system**. This questionnaire collects information on the existence and the characteristics of the behavioural surveillance system as a whole in a given country. Click here for file

## References

[B1] European Centre for Disease Prevention and Control/WHO Regional Office for Europe HIV/AIDS surveillance in Europe 2007 2008 Stockholm

[B2] European Centre for Disease Prevention and Control (ECDC) Migrant health: Epidemiology of HIV and AIDS inmigrant communities and ethnic minorities in EU/EEA countries. ECDC technical report 2009 Stockholm

[B3] European Centre for Disease Prevention and Control Annual Epidemiological Report on Communicable Diseases in Europe 2008 2008 Stockholm 22114980

[B4] Fenton K Lowndes CM The European Surveillance of Sexually Transmitted Infections (ESSTI) Network Recent trends in the epidemiology of sexually transmitted infections in the European Union Sex Transm Infect 2004 80 255 263 10.1136/sti.2004.009415 15295121 PMC1744866

[B5] Righarts AA Simms I Wallace L Solomou M Fenton KA Syphilis surveillance and epidemiology in the United Kingdom Eurosurveillance 2004 9 21 25 15677851

[B6] Marcus U Bremer V Hamouda O Kramer MH Freiwald M Jessen H Rausch M Reinhardt B Rothaar A Schmidt W Understanding recent increases in the incidence of sexually transmitted infections in men having sex with men: changes in risk behavior from risk avoidance to risk reduction Sex Transm Dis 2006 33 11 17 10.1097/01.olq.0000187224.10428.31 16385216

[B7] Ward H Martin I Macdonald N Alexander S Simms I Fenton K Lymphogranuloma venereum in the United Kingdom Clin Infect Dis 2007 44 26 32 10.1086/509922 17143811

[B8] Urbanus AT van de Laar TJ Stolte IG Schinkel J Heijman T Coutinho RA Prins M Hepatitis C virus infections among HIV-infected men who have sex with men: an expanding epidemic AIDS 2009 23 F1 F7 10.1097/QAD.0b013e32832e5631 19542864

[B9] Mertens T Carael M Sato P Cleland J Ward H Smith GD Prevention indicators for evaluating the progress of national AIDS programmes (Editorial Review) AIDS 1994 8 1359 1369 10.1097/00002030-199410000-00002 7818807

[B10] Dubois-Arber F Jeannin A Spencer B Long term global evaluation of a national AIDS prevention strategy: The case of Switzerland AIDS 1999 13 2571 2582 10.1097/00002030-199912240-00011 10630527

[B11] Bozon M Doré V Laporte A Lert F Meyer L Théry I Pelé G Sobel A Souteyrand Y Evaluer la prévention de l'infection par le VIH en France: synthèse des données quantitiatives, 1994-1999 Agence nationale de recherches sur le sida (ANRS) 1999

[B12] Centre d'estudis epidemiològics sobre l'HIV/sida de Catalunya (CEESCAT) Sistema integrat de vigilancia epidemiologica de Sida/VIH/ITS a Catalunya (SIVES): Informe bianual Barcelona: Generalitat de Catalunya, Departament de Salut 2008

[B13] Brown AE Tomkins S Logan L LaMontagne D Munro H Hope VD Righarts A Blackham J Rice B Chadborn T Monitoring the effectiveness of HIV and STI prevention initiatives in England, Wales, and Northern Ireland: where are we now? Sex Transm Infect 2006 82 4 10 10.1136/sti.2005.016386 16461593 PMC2563811

[B14] Bochow M Chiarotti F Davies P Dubois-Arber F Dür W Fouchard J Gruet F McManus T Markert S Sandfort T Sexual behaviour of gay and bisexual men in eight European countries AIDS Care 1994 6 533 549 10.1080/09540129408258668 7711087

[B15] Bochow M Rosenbrock R, Wright MT The response of gay german men to HIV: the national gay press surveys, 1987-96 Partnership and Pragmatism: germany's response to AIDS prevention and care 2001 London: Routledge 129 42

[B16] Bochow M Jauffret M Michel A Schiltz M-A Broqua C, Lert F, Souteyrand Y Les évolutions des comportements sexuels et les modes de vie à travers les enquêtes réalisées dans la presse gaie en France (1985-2000) Homosexualités au temps du sida: tension sociales et identitaires 2003 Paris: Agence nationale de recherche sur le sida 35 54

[B17] Elford J Bolding G Sherr L High-risk sexual behaviour increases among London gay men between 1998 and 2001: what is the role of HIV optimism? AIDS 2002 16 1537 1544 10.1097/00002030-200207260-00011 12131192

[B18] Emmanuelli J Desenclos JC Harm reduction interventions, behaviours and associated health outcomes in France, 1996-2003 Addiction 2005 100 1690 1700 10.1111/j.1360-0443.2005.01271.x 16277629

[B19] Hope VD Rogers PA Jordan L Paine T Barnett S Parry J Sustained increase in the sharing of needles and syringes among drug users in England and Wales AIDS 2002 16 2494 2496 10.1097/00002030-200212060-00022 12461431

[B20] Benninghoff F Morency P Geense R Huissoud T Dubois-Arber F Health trends among drug users attending needle exchange programmes in Switzerland (1994-2000) Aids Care-Psychological and Socio-Medical Aspects of Aids/Hiv 2006 18 371 375 10.1080/09540120500429018 16809115

[B21] Johnson AM Mercer CH Erens B Copas AJ McManus S Wellings K Fenton KA Korovessis C Macdowall W Nanchahal K Sexual behaviour in Britain: partnerships, practices, and HIV risk behaviours Lancet 2001 358 1835 1842 10.1016/S0140-6736(01)06883-0 11741621

[B22] Dubois-Arber F Jeannin A Konings E Paccaud F Increased condom use without other major changes in sexual behavior among the general population in Switzerland Am J Public Health 1997 87 558 566 10.2105/AJPH.87.4.558 9146432 PMC1380833

[B23] de Vroome EM Paalman ME Dingelstad AA Kolker L Sandfort TG Increase in safe sex among the young and non-monogamous: knowledge, attitudes and behavior regarding safe sex and condom use in The Netherlands from 1987 to 1993 Patient Education & Counseling 1994 24 279 288 10.1016/0738-3991(94)90071-x 7753721

[B24] Ward H Day S Weber J Risky business: health and safety in the sex industry over a 9 year period Sex Transm Infect 1999 75 340 343 10.1136/sti.75.5.340 10616360 PMC1758230

[B25] Sethi G Holden BM Gaffney J Greene L Ghani AC Ward H HIV, sexually transmitted infections and risk behaviours in male sex workers in London over a 10 year period Sex Transm Infect 2006 82 359 363 10.1136/sti.2005.019257 16916883 PMC2563850

[B26] Pisani E Brown T Saidel T Rehle T Carael M Meeting the Behavioural Data Collection Needs of National HIV/AIDS and STD Programmes. A joint Impact/FHI/UNAIDS workshop: report and conclusions 1998

[B27] Behavioral surveillance surveys: guidelines for repeated behavioral surveys in populations at risk of HIV 2000 Arlington, VA: FHI

[B28] UNAIDS/WHO-Working Group on Global HIV/AIDS and STI Surveillance Guidelines for second generation HIV surveillance 2000 Geneva: UNAIDS/WHO

[B29] UNAIDS/WHO Working Group on Global HIV/AIDS and STD Surveillance The pre-surveillance assessment: guidelines for planning serosurveillance of HIV, prevalence of sexually transmitted infections and the behavioural components of second generation surveillance of HIV. Geneva 2005

[B30] Garnett G Garcia-Calleja J Rehle T Gregson S Behavioural data as an adjunct to HIV surveillance data Sex Transm Infect 2006 82 57 62 10.1136/sti.2005.016543 PMC2593068 16581762

[B31] McGarrigle CA Fenton KA Gill ON Hughes G Morgan D Evans B Behavioural surveillance: the value of national coordination Sex Transm Infect 2002 78 398 405 10.1136/sti.78.6.398 12473798 PMC1758341

[B32] UNAIDS/WHO-Working Group on Global HIV/AIDS and STI Surveillance Initiating second generation HIV surveillance systems: practical guidelines 2002 Geneva: UNAIDS/WHO

[B33] Monitoring the Declaration of Commitment on HIV/AIDS: guidelines on construction of core indicators: 2010 reporting 2009 Geneva: UNAIDS

[B34] European Monitoring Centre for Drugs and Drug Addiction An overview of the drug-related infectious diseases (DRID) key indicator 2009 http://www.emcdda.europa.eu/attachements.cfm/att_67055_EN_EMCDDA-DRID-overview.pdf. http://www.emcdda.europa.eu/attachements.cfm/att_67055_EN_EMCDDA-DRID-overview.pdf 28-10-2009

[B35] Dubois-Arber F Jeannin A Meystre-Agustoni G Un système de surveillance de deuxième génération pour améliorer la surveillance du VIH/sida en Suisse Bulletin OFSP 2006 15 277 281

[B36] Diaz T Garcia-Calleja J Ghys PD Sabin K Advances and future directions in HIV surveillance in low- and middle-income countries Curr Opin HIV AIDS 2009 4 253 259 10.1097/COH.0b013e32832c1898 19532061

[B37] Sullivan PSGE HIV behavioral surveillance Public Health Rep 2007 122 1 79 10.1177/00333549071220S104 PMC1804114 17354523

[B38] Ghys P Walker N Ward H Miller RE Improved methods and tools for HIV/AIDS estimates and projections Sex Transm Infect 2006 82 189 266 10.1136/sti.2006.021030 18647859 PMC2569833

[B39] Cleland J Boerma JT Carael M Weir SSE Measurement of sexual behaviour Sex Transm Infect 2004 80 423 546 10.1136/sti.2004.013151 15572634 PMC1765850

[B40] Schwartländer B Coutinho R Loures LE The HIV/AIDS epidemic in the Latin America and Carribean Region AIDS 2002 16 S1 S82 10.1097/00002030-200212003-00001

[B41] European Centre for Disease Prevention and Control (ECDC) Mapping of HIV/STI behavioural surveillance in Europe. Technical report 2009 Stockholm

[B42] Elford J Jeannin A Spencer B Gervasoni J-P van de Laar MJ Dubois-Arber F the HIV & STI Behavioural Surveillance Mapping Group HIV and STI behavioural surveillance among men who have sex with men (MSM) in Europe Eurosurveillance 2009 14 19941807 10.2807/ese.14.47.19414-en

[B43] UNAIDS A framework for monitoring and evaluating HIV prevention programmes for most-at-risk populations 2007 Geneva: UNAIDS

[B44] Vernazza P Hirschel B Bernasconi E Flepp M HIV- infizierte Menschen ohne andere STD sind unter wirksamer antiretroviraler Therapie sexuell nicht infektiös [HIV-infected people free of other STDs are sexually not infectious on effective antiretroviral therapy] Schweiz Arzteztg 2008 89 165 169

[B45] European Centre for Disease Prevention and Control (ECDC) Migrant health: Access to HIV prevention, treatment and care for migrant populations in EU/EEA countries. ECDC technical report 2009 Stockholm

[B46] Aceijas C Hickman M Donoghoe MC Burrows D Stuikyte R Access and coverage of needle and syringe programmes (NSP) in Central and Eastern Europe and Central Asia Addiction 2007 102 1244 1250 10.1111/j.1360-0443.2007.01848.x 17565564

