# Supplementary material for: Mapping HIV/STI behavioural surveillance in Europe
Source: BMC Infect Dis. 2010 Oct 4;10:290. doi: 10.1186/1471-2334-10-290 (PMC2959062; doi:10.1186/1471-2334-10-290)
Supplement: Additional file 1 — questionnaire related to behavioural surveillance in the general population. This questionnaire collects information on the existence and the characteristics of behavioural surveillance conducted in the general population in a given country. [file 1471-2334-10-290-S1.DOC]

| 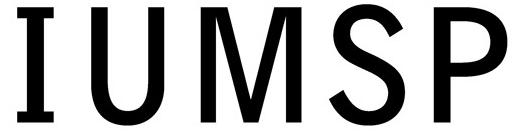  Institut universitaire de médecine sociale et préventive | Rue du Bugnon 17 CH-1005 Lausanne Tél. +41 21 314 72 72 Fax +41 21 314 73 73 www.iumsp.ch |  | 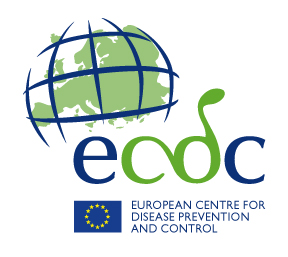 |
| --- | --- | --- | --- |

ECDC-mandated 2008 survey of Behavioural Surveillance related to HIV and STI
in European Union member states and other countries

General Population questionnaire

**What is this questionnaire ?**

This questionnaire is part of the survey of behavioural surveillance programmes related to HIV and STI in the EU and other countries undertaken on behalf of the European Centre for Disease Control (ECDC) by an international team of specialists led by the Institute of Social and Preventive Medicine (IUMSP), University Hospital Centre and University of Lausanne, Lausanne, Switzerland.

The survey is divided into nine questionnaires. The questionnaires are in English. This "General Population questionnaire", addresses the existence, scope and functioning of behavioural surveillance in the general population.

There are eight other questionnaires: one addresses behavioural surveillance as a system and the seven others are population-specific: young people, men having sex with other men, injecting drug users, sex workers, STI clinics clients, people living with HIV/AIDS, ethnic minorities and migrants. These questionnaires are to be sent to the appropriate designated people.

A **glossary** of terms is provided at the end of this questionnaire.

**Who should complete this questionnaire ?**

This questionnaire should be completed by the most appropriate person for this field. This will generally be the person responsible for behavioural surveillance programmes in the general population, or someone involved in, or well-informed about, these activities. The questionnaire is provided as a computer file and can be forwarded to as many people as necessary (see below: "How to complete the questionnaire").

**How to complete this questionnaire**

The questionnaire is provided as a computer file: a Microsoft Word 2000 form containing three types of fields. The boxed Tick fields are filled by clicking with the mouse; the Comment fields are text fields that will expand as needed to accommodate any amount of text; in the Drop-down lists, only one option can be chosen. If you need to give more information, please write this in the 'Comments' field.

When using acronyms, be sure to include the unabbreviated form of the expression when the acronym is first used.

It may be necessary to obtain the required information from different people. If the questionnaire is transmitted to several people, please make sure this is done sequentially, so that the responses from different people are entered in one file only. One single completed questionnaire file, combining all responses, should be sent back to the IUMSP team.

Some questions request published or unpublished scientific articles, reports or other documents. If at all possible, provide these in electronic form and send them by email to the IUMSP team. If no electronic version exists, please send us a paper copy by postal mail.

Some of the questions require expressing a judgement or opinion. Please answer these to the best of your knowledge.

**Where and how to return the completed questionnaire and other information**

The completed questionnaire and other electronic documents are to be returned by email to **andre.jeannin@chuv.ch**

The postal address for paper documents is: IUMSP, attn: A. Jeannin, rue du Bugnon 17, CH 1005 Lausanne, Switzerland.

**Please complete the questionnaire and return it by August 15th, 2008.**

**Contact persons at the IUMSP**

| André Jeannin  andre.jeannin@chuv.ch  Tel. +44 21 314 7296 | Brenda Spencer  brenda.spencer@chuv.ch  Tel. +44 21 314 7297 | Françoise Dubois-Arber  francoise.dubois-arber@chuv.ch  Tel. +44 21 314 7290 |
| --- | --- | --- |

|  |  |
| --- | --- |

**The completed questionnaire is to be sent back to this person, who is responsible for completing this General Population questionnaire:**

| Institution  First name, Family name  E-mail  Postal address  Phone number |  |
| --- | --- |

**If other persons participated in completing the questionnaire, please provide their first name, family name, institution, email and telephone in case the research team needs some clarification:**

| Person 1 |  |
| --- | --- |
| Person 2 |  |
| Person 3 |  |

***Thank you very much to all who provided information for this survey !***

In this questionnaire, we focus on behavioural (as opposed to biological) surveillance) in the general population as a system. We understand a 'behavioural surveillance system' to be the collection and use of data from different sources and/or different time points to globally ascertain the state and evolution of the HIV/Aids and/or STI epidemics at the behavioural, as opposed to biological, level.

| **A** | Behavioural surveillance in the General population as a system *We understand as 'behavioural surveillance system' the collection and use of data from different sources and/or different time points to globally ascertain the state and evolution of the HIV/Aids and/or STI epidemics at the behavioural, as opposed to biological, level.* | | |
| --- | --- | --- | --- |
| 1 | In your view, is there a system of behavioural surveillance of the general population in your country ? | yes  no | Comment: |
| 2 | If there is such a system in your country, since what year has it been in operation ? | since | Comment: |

| **B** | Topics followed as part of behavioural surveillance in the General population *Here we want to know whether the following broad themes are included in the surveillance, whatever the data collection method. These do not refer to specific items, each topic may include many items, with various wordings, etc. We do not ask about obvious demographic characteristics such as gender or age. For each line, please answer whether the topic is monitored regularly, irregularly or not monitored as part of the surveillance.* | | | |
| --- | --- | --- | --- | --- |
|  |  | **Regularly Irregularly Not monitored** | **Comments** | |
| **1** | **Knowledge and attitudes** |  |  | |
|  | Attitudes towards PLWHA |  |  | |
|  | Knowledge about HIV/Aids infection and/or treatments |  |  | |
|  | Knowledge about STI infection and/or treatments |  |  | |
|  | Awareness of prevention activities |  | |  |
| **2** | Sexual Relationships and sexual partners |  | |  |
|  | Types of partners/relationships, such as regular partner, casual partners |  | |  |
|  | Concurrency |  | |  |
| **3** | Sexual activity and lifestyle |  | |  |
|  | Sexual orientation |  | |  |
|  | Sexual activity, such as number of partners, frequency of sexual contacts |  | |  |
|  | Contraception |  | |  |
|  | Recourse to prostitution (as client) |  | |  |
|  | Recourse to prostitution (as sex worker) |  | |  |
|  | How and where partners are met |  | |  |
|  | Sexual practices |  | |  |
| **4** | Exposure to risk of infection |  | |  |
|  | Condom use at last intercourse |  | |  |
|  | Condom use with different types of partners |  | |  |
|  | Condom use in different types of sexual practice (e.g. vaginal, anal, oral sex) |  | |  |
|  | Disclosure of HIV status to sexual partners |  | |  |
| **5** | HIV and other STIs |  | |  |
|  | HIV testing |  | |  |
|  | Result of HIV test (self-reported) |  | |  |
|  | Result of HIV test (measured) |  | |  |
|  | Current or past STIs other than HIV and Hepatitis |  | |  |
|  | Hepatitis B vaccine |  | |  |
|  | Hepatitis B status (self-reported) |  | |  |
|  | Hepatitis B status (measured) |  | |  |
| **6** | Drugs and substance use |  | |  |
|  | Types of drugs consumed |  |  | |
|  | Injecting drug use |  |  | |
|  | Use of psycho-active substances (including alcohol) and intercourse |  |  | |
| **7** | Health and access to care |  |  | |
|  | Antiretroviral treatment |  |  | |
|  | Access to care and support |  |  | |
| **8** | Sociodemographic characteristics |  |  | |
|  | Education |  |  | |
|  | Employment |  |  | |
|  | Nationality and/or ethnic origin |  |  | |
|  | Housing conditions |  |  | |
| 9 | Other topics followed (please specify in Comments) |  |  | |

**Questions D and E deal with sources of data used for HIV/Aids and STI behavioural surveillance *in the general population* since the start of the HIV/Aids epidemic in 1985. We inquire first about data collected in a repeated way, actual or intended (Question D) and then about one-off studies (Question E). If a one-off study is actually the first in a planned series, please mention it as a repeated study. Please mention only those sources of data that are relevant for behavioural surveillance in the general population.**

| **D1** | Repeated cross-sectional surveys, cohorts and other routine or repeated data collection relevant for HIV/Aids and STI behavioural surveillance in the general population The focus is on the national studies. Mention the lower-scale studies if they are important for surveillance according to your judgement. Please provide information for each of the series of cross-sectional surveys, cohorts or repeated routine data collection (up to a maximum of 10). Start with the most recent series and work backward in time. If important characteristics (design, target population, sample size, etc.) of the series have changed over time, use one line for each. Use the Comment field to provide details as needed. See example at bottom of table. |
| --- | --- |

|  | **Name of survey or** | **Institution responsible** | **Years done** | **Tick and give year if planned to conti-nue** | **Target population** | **Design** | **Mode of administration** | **Average sample size** | **Coverage** | **Comment** |
| --- | --- | --- | --- | --- | --- | --- | --- | --- | --- | --- |
| 1 |  |  |  |  |  |  |  | 0 |  |  |
| 2 |  |  |  |  |  |  |  | 0 |  |  |
| 3 |  |  |  |  |  |  |  | 0 |  |  |
| 4 |  |  |  |  |  |  |  | 0 |  |  |
| 5 |  |  |  |  |  |  |  | 0 |  |  |
| 6 |  |  |  |  |  |  |  | 0 |  |  |
| 7 |  |  |  |  |  |  |  | 0 |  |  |
| 8 |  |  |  |  |  |  |  | 0 |  |  |
| 9 |  |  |  |  |  |  |  | 0 |  |  |
| 10 |  |  |  |  |  |  |  | 0 |  |  |
| ***Example for Switzerland*** | | |  |  |  |  |  |  |  |  |
|  | *EPSS* | *Institute Of Social and Preventive Medicine, Lausanne* | *1989, 1990, 1991, 1992, 1994, 1997, 2000, 2007* |  | *General population aged 17-45* | *repeated cross-sectional* | *telephone interview* | *2800* | *national* | *Expected to be repeated, but year not known* |
|  | *EPSS* | *Institute Of Social and Preventive Medicine, Lausanne* | *jan. 1987, oct. 1987, 1988* |  | *General population aged 17-30* | *repeated cross-sectional* | *telephone interview* | *1200* | *national (Ticino not included)* |  |

| **D2** | Additional information regarding studies mentioned in Table D1 In this table, please provide additional information about each of the series of study mentioned in Table D1. The name of the study should refer to one of the series of studies in Table D1. Use the 'Comment' field for any additional information you might want to provide. | | |
| --- | --- | --- | --- |
| **1** | **Name of the study :** | Institution in charge: address, telephone, email, internet site, etc. : | Contact person: |
|  | References or Internet links to the survey protocol if available: | References to published results if available: | Additional information or comment: |
| **2** | **Name of the study :** | Institution in charge: address, telephone, email, internet site, etc. : | Contact person: |
|  | References or Internet links to the survey protocol if available: | References to published results if available: | Additional information or comment: |
| **3** | **Name of the study :** | Institution in charge: address, telephone, email, internet site, etc. : | Contact person: |
|  | References or Internet links to the survey protocol if available: | References to published results if available: | Additional information or comment: |
| **4** | **Name of the study :** | Institution in charge: address, telephone, email, internet site, etc. : | Contact person: |
|  | References or Internet links to the survey protocol if available: | References to published results if available: | Additional information or comment: |
| **5** | **Name of the study :** | Institution in charge: address, telephone, email, internet site, etc. : | Contact person: |
|  | References or Internet links to the survey protocol if available: | References to published results if available: | Additional information or comment: |
| **6** | **Name of the study :** | Institution in charge: address, telephone, email, internet site, etc. : | Contact person: |
|  | References or Internet links to the survey protocol if available: | References to published results if available: | Additional information or comment: |
| **7** | **Name of the study :** | Institution in charge: address, telephone, email, internet site, etc. : | Contact person: |
|  | References or Internet links to the survey protocol if available: | References to published results if available: | Additional information or comment: |
| **8** | **Name of the study :** | Institution in charge: address, telephone, email, internet site, etc. : | Contact person: |
|  | References or Internet links to the survey protocol if available: | References to published results if available: | Additional information or comment: |
| **9** | **Name of the study :** | Institution in charge: address, telephone, email, internet site, etc. : | Contact person: |
|  | References or Internet links to the survey protocol if available: | References to published results if available: | Additional information or comment: |
| **10** | **Name of the study :** | Institution in charge: address, telephone, email, internet site, etc. : | Contact person: |
|  | References or Internet links to the survey protocol if available: | References to published results if available: | Additional information or comment: |

| **E** | | Design of the one-off surveys or studies relevant for behavioural surveillance among THE GENERAL POPULATION Provide information for each of the one-off surveys or studies (up to a maximum of 20), starting with the most recent one and working backward in time. Repeated surveys, cohorts and repeated routine data collection are dealt with in the previous question. | | | | | | |
| --- | --- | --- | --- | --- | --- | --- | --- | --- |
|  | **Name of survey** | **Year** | **Target population** | **Sampling approach** | **Mode of administration** | **Total N** | **Coverage** | **Comment** |
| 1 |  |  |  |  |  | 0 |  |  |
| 2 |  |  |  |  |  | 0 |  |  |
| 3 |  |  |  |  |  | 0 |  |  |
| 4 |  |  |  |  |  | 0 |  |  |
| 5 |  |  |  |  |  | 0 |  |  |
| 6 |  |  |  |  |  | 0 |  |  |
| 7 |  |  |  |  |  | 0 |  |  |
| 8 |  |  |  |  |  | 0 |  |  |
| 9 |  |  |  |  |  | 0 |  |  |
| 10 |  |  |  |  |  | 0 |  |  |
| 11 |  |  |  |  |  | 0 |  |  |
| 12 |  |  |  |  |  | 0 |  |  |
| 13 |  |  |  |  |  | 0 |  |  |
| 14 |  |  |  |  |  | 0 |  |  |
| 15 |  |  |  |  |  | 0 |  |  |
| 16 |  |  |  |  |  | 0 |  |  |
| 17 |  |  |  |  |  | 0 |  |  |
| 18 |  |  |  |  |  | 0 |  |  |
| 19 |  |  |  |  |  | 0 |  |  |
| 20 |  |  |  |  |  | 0 |  |  |

| **F** | **Main indicators followed as part of behavioural surveillance among the general population**  Here we inquire about the main indicators according to your judgement. In general, these will be drawn from repeated data collection sources. Provide detailed information for the numerator and denominator, including subgroups concerned, reference periods and names of data sources (see example for Switzerland). | | | | |
| --- | --- | --- | --- | --- | --- |
|  | **Name/Interpretation of indicator and remarks/comments** | **Name of data source** | **Numerator** | **Denominator** | **Followed in current form since (year)** |
| a |  |  |  |  |  |
| b |  |  |  |  |  |
| c |  |  |  |  |  |
| d |  |  |  |  |  |
| e |  |  |  |  |  |
| f |  |  |  |  |  |
| g |  |  |  |  |  |
| h |  |  |  |  |  |
| i |  |  |  |  |  |
| j |  |  |  |  |  |
| k |  |  |  |  |  |
| l |  |  |  |  |  |
| m |  |  |  |  |  |
| n |  |  |  |  |  |
| o |  |  |  |  |  |
|  | ***Example for Switzerland*** |  |  |  |  |
|  | *Consistent condom use with casual partners: interpreted as indicator of evolution of potential risk of infection in the general population* | *repeated telephone survey for the evaluation of Aids prevention in Switzerland (EPSS)* | *number of people aged 17 to 30 years who consistently ("always") used condom with their casual partners in the last 6 months* | *number of people aged 17 to 30 years who had one or more casual partners in the last 6 months* | *1987* |

| If you want to comment about this questionnaire, please do it: |  |
| --- | --- |

**This is the end of the General Population Surveillance questionnaire.**

**See return instruction on Page 2**

**Thank you very much for your participation!**

**Glossary**

As we need to ask information about behavioural surveillance in many different countries, we give broad and general operational definitions to make clear what information we are seeking.

| **Behavioural surveillance system** | We understand as 'behavioural surveillance system' the collection and use of data from different sources and/or different time points to globally ascertain the state and evolution of the HIV/Aids and/or STI epidemics at the behavioural, as opposed to biological, level. |
| --- | --- |
| **Concurrency** | A concurrent partnership is a sexual partnership in which one or more of the members has other sexual partners, with repeated sexual activity with at least the original partner. [Gorbach PM, et al., 2002] |
| **Judgement or opinion** | Some of the questions require expressing a judgement or opinion. We want to know what is the situation according to the best of your knowledge, even in absence of definitive information. |
| **Sexual contacts** | We use 'sexual contacts' as a broad term to refer to intercourse or other sexual acts. Their contents may not be the same in different populations. |
| **Sustainability** | In the context of behavioural and second generation surveillance systems, we understand 'sustainability' as the ability for the surveillance system to perform its functions over time. This is dependent upon the availability of appropriate resources, such as financing, expertise, political commitment, etc. |
| **WHO recommendations for "Second Generation Surveillance System" in low-level and concentrated epidemics** [Source: UNAIDS/WHO - Working Group on Global HIV/AIDS and STI Surveillance. Guidelines for second generation HIV surveillance. Geneva: UNAIDS/WHO; 2000] | **Recommendations for surveillance in a low-level epidemic**  • Cross-sectional surveys of behaviour in sub-populations with risk behaviour  • Surveillance of STIs and other biological markers of risk  • HIV surveillance in sub-populations at risk  • HIV and AIDS case reporting  • Tracking of HIV in donated blood  **Recommendations for surveillance in a concentrated epidemic**  HIV surveillance in a concentrated epidemic will contain all of the elements recommended for a low-level epidemic, but will add elements that focus more on the intersection between groups with different levels of risk.  • HIV and behavioural surveillance in sub-populations with risk behaviour  • HIV and behavioural surveillance in bridging groups  • Cross-sectional surveys of behaviour in the general population  • HIV sentinel surveillance in the general population, urban areas |
