# Supplementary material for: Mapping HIV/STI behavioural surveillance in Europe
Source: BMC Infect Dis. 2010 Oct 4;10:290. doi: 10.1186/1471-2334-10-290 (PMC2959062; doi:10.1186/1471-2334-10-290)
Supplement: Additional file 2 — questionnaire related to the behavioural surveillance system. This questionnaire collects information on the existence and the characteristics of the behavioural surveillance system as a whole in a given country. [file 1471-2334-10-290-S2.DOC]

| 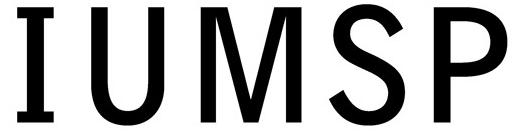  Institut universitaire de médecine sociale et préventive | Rue du Bugnon 17 CH-1005 Lausanne Tél. +41 21 314 72 72 Fax +41 21 314 73 73 www.iumsp.ch |  | 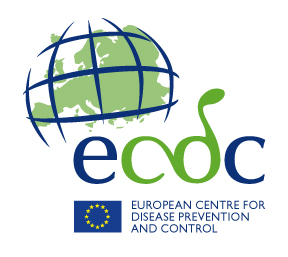 |
| --- | --- | --- | --- |

ECDC-mandated 2008 survey of Behavioural Surveillance related to HIV and STI
in European Union member states and other countries

Surveillance System questionnaire

**What is this questionnaire ?**

This questionnaire is part of the survey of behavioural surveillance programmes related to HIV and STI in EU and other countries undertaken for the European Centre for Disease Control (ECDC) by an international team of specialists led by the Institute of Social and Preventive Medicine (IUMSP), University Hospital Centre and University of Lausanne, Lausanne, Switzerland.

The survey is divided into nine questionnaires. The questionnaires are in English. The present one, the "Surveillance System questionnaire", addresses the existence, scope and functioning of the surveillance system and is comprised of two sections: Section 1) the Behavioural Surveillance System (questions A to C), and Section 2) the Second Generation Surveillance System integrating biological surveillance and behavioural surveillance (questions D to E). In both sections, the focus is on the system level.

The other eight questionnaires are population-specific: general population, young people, men having sex with other men, injecting drug users, sex workers, STI clinics attendees, people living with HIV/AIDS, ethnic minorities and migrants. These questionnaires are to be sent to the appropriate designated people.

A **glossary** of terms is provided at the end of this questionnaire.

**Who should complete this questionnaire ?**

This questionnaire should be completed by the most relevant person. This will generally be the person responsible for the behavioural surveillance programmes related to HIV and STI, or someone involved in or well-informed about these activities. The questionnaire is provided as a computer file and can be transmitted to as many people as necessary (see below: "How to complete the questionnaire").

**How to complete this questionnaire ?**

The questionnaire is provided as a computer file: a Microsoft Word 2000 form containing three types of fields. The Tick fields are filled by clicking with the mouse; the Comment fields are text fields that will expand as needed to accommodate any amount of text; in the Drop-down lists, only one option can be chosen. If you need to give more information, please write this in the 'Comments' field.

When using acronyms, be sure to include the unabbreviated form of the expression when the acronym is first used.

It may be necessary to get the needed information from different people. The questionnaire can be transmitted to as many people as necessary. If that is the case, please make sure this is done sequentially and that the responses from the different people are entered in only one file. Only one questionnaire file, containing all the responses, should be sent back to the IUMSP team.

Some questions request published or unpublished scientific articles, reports or other documents. If at all possible, provide these in electronic form and send them by email to the IUMSP team. If no electronic version exists, please send us a paper copy by postal mail.

Some of the questions require judgements. Please answer these to the best of your knowledge.

**Where and how to return the completed questionnaire and other information ?**

The completed questionnaire and other electronic documents are to be returned by email to andre.jeannin@chuv.ch

The postal address for paper documents is: IUMSP, attn A. Jeannin, rue du Bugnon 17, CH 1005 Lausanne, Switzerland.

**Please complete the questionnaire and return it by August 15th, 2008.**

**Contact persons at the IUMSP**

| André Jeannin  andre.jeannin@chuv.ch  Tel. +44 21 314 7296 | Brenda Spencer  brenda.spencer@chuv.ch  Tel. +44 21 314 7297 | Françoise Dubois-Arber  francoise.dubois-arber@chuv.ch  Tel. +44 21 314 7290 |
| --- | --- | --- |

Thank you very much for your collaboration – it is greatly appreciated!

|  |  |
| --- | --- |

Person responsible for completing the Surveillance System questionnaire

| Institution  First name, name  E-mail  Postal address  Phone number |  |
| --- | --- |

Thank you very much for taking part to this survey.

**Section 1 - Behavioural Surveillance System**

We focus on behavioural surveillance in various populations as a system: regularly or not regularly repeated surveys or studies or other data collection methods on HIV/Aids-related behaviours, the results of which are used to ascertain the state and evolution of the HIV/AIDS and/or STI epidemics in your country.

| **A** | Behavioural surveillance as a system | | |
| --- | --- | --- | --- |
| 1 | According to your judgement, is there a system of behavioural surveillance in your country ? | Yes  No | Comments: |
| 2 | If there is such a system in your country, since what year has it been operating ? | since | Comments: |

| **B** | Populations covered by the behavioural surveillance system *For each line, please answer whether the population is monitored or not monitored as part of the surveillance..* | | |
| --- | --- | --- | --- |
|  |  | **Monitored Not monitored** | **Comments** |
|  | General Population |  |  |
|  | Young people |  |  |
|  | Men having Sex with other Men |  |  |
|  | Injecting Drug Users |  |  |
|  | Sex Workers |  |  |
|  | STI clinics attenders |  |  |
|  | People Living With HIV/Aids |  |  |
|  | Ethnic Minorities and Migrants |  |  |
|  | Other (please specify in Comments field) |  |  |

| **C** | Functioning of the Behavioural Surveillance System | |
| --- | --- | --- |
| 1 | Are there documents establishing or describing the existence and/or functioning of the Behavioural Surveillance System? Please indicate the references and, if possible, send or mail us these documents | References of documents: |
| 2 | Is there a centralized body for the management and/or coordination of the Behavioural Surveillance System ? Please indicate its name. | Name of the body or agency: |
| 3 | If a full or partial Behavioural Surveillance System currently exists in your country, since when is it operational ? | Since year       Comment: |
| 4 | How is the Behavioural Surveillance System currently financed in your country ? Please specify whether financing come from the national government or administration, international agencies, national or international grants from foundations, etc. | Financing from (tick all that apply):  National government/administration  International agencies Grants from foundations  Research grants  Other. Specify: |
| 5 | According to your judgement, what is the sustainability within a five-year time frame (about 2012) of the Behavioural Surveillance System presently operational in your country? (feel free to add comments if necessary) | Sustainability is  Comment: |
| 6 | Are there published analyses, articles or reports concerning the Behavioural Surveillance System as such ? Please indicate the references and, if possible, send or mail us these documents. | References of documents: |

**Section 2 - Second Generation Surveillance System**

We focus on Second Generation surveillance as a system, including both a biological surveillance component and a behavioural surveillance component (regularly or not regularly repeated surveys or studies or other data collection methods on HIV/Aids-related behaviours), the integrated results of which are used to ascertain the state and evolution of the HIV/AIDS and/or STI epidemics in your country.

| **D** | Second Generation surveillance as a system | | |
| --- | --- | --- | --- |
| 1 | According to your judgement, is a Second Generation Surveillance System presently operational in your country? (feel free to add comments if necessary) | Yes, fully operational  Yes, partly operational  No | Comments: |
| 2 | If there is such a system in your country, since what year has it been operating ? | since | Comments: |

| **E** | Functioning of the Second Generation Surveillance System | | |
| --- | --- | --- | --- |
| 1 | Are there documents establishing or describing the existence and/or functioning of the Second Generation Surveillance System? Please indicate the references and, if possible, send or mail us these documents | References of documents: | |
| 2 | Is there a centralized body for the management and/or coordination of the Second Generation Surveillance System ? Please indicate its name. | Name of the body or agency: | |
| 3 | If a full or partial Second Generation Surveillance System currently exists in your country, since when is it operational ? | Since year       Comment: | |
| 4 | How is the Second Generation Surveillance System currently financed in your country ? Please specify whether financing come from the national government or administration, international agencies, national or international grants from foundations, etc. | Financing from (tick all that apply):  National government/administration  International agencies Grants from foundations  Research grants  Other. Specify: | |
| 5 | This question is about the analysis and interpretation of the system-wide information provided by the Second Generation Surveillance system in your country. Is the integration of information done system-wide in a formalized way ? | Tick one:  Formalized, with a specific entity responsible for this task  Provided less formally by a network of institutions  Deficient or non-existing | Please describe briefly how this is done: |
| 6 | This question is about the diffusion of the system-wide information provided by the Second Generation Surveillance system in your country. Please describe briefly how this is done. |  | |
| 7 | Please describe briefly how the Second Generation Surveillance system works in your country. We refer here to the interplay and/or integration of the biological and behavioural components. |  | |
| 8 | How is the Second Generation Surveillance System currently used in your country ? | Tick all that apply:  Used as an advocacy tool for increase resources and expanded responses  Assist in the targeting and evaluation of overall prevention and care programmes  Indicators of progress for national programmes  Building support for continued prevention and care efforts  Interpreting trends in HIV incidence or prevalence  Programme planning  Identify the drivers of the epidemic  To project future prevention and care needs  Monitoring and evaluation  Other: | |
| 9 | According to your judgement, what is the sustainability within a five-year time frame (about 2012) of the Second Generation Surveillance System presently operational in your country? (feel free to add comments if necessary) | Sustainability is  Comment: | |
| 10 | Are there published analyses, articles or reports concerning the Second Generation Surveillance System as such ? Please indicate the references and, if possible, send or mail us these documents. | References of documents: | |
| 11 | This question deals with problems or barriers to the sustainability of the Second Generation Surveillance system. Please describe the problems and barriers you see in establishing such a system if it does not yet exists in your country, or to its sustainability if it exists. |  | |
| 12 | Do you have one or more examples of positive experiences resulting from the existence of a Second Generation Surveillance system in your country ? |  | |

**This is the end of the Surveillance System questionnaire.**

**See return instruction on Page 2**

**Thank you very much for your participation!**

**Glossary**

As we need to ask information about behavioural surveillance in many different countries, we give broad and general operational definitions to make clear what information we are seeking.

| **Behavioural surveillance system** | We understand as 'behavioural surveillance system' the collection and use of data from different sources and/or different time points to globally ascertain the state and evolution of the HIV/Aids and/or STI epidemics at the behavioural, as opposed to biological, level. |
| --- | --- |
| **Concurrency** | A concurrent partnership is a sexual partnership in which one or more of the members has other sexual partners, with repeated sexual activity with at least the original partner. [Gorbach PM, et al., 2002] |
| **Judgement or opinion** | Some of the questions require expressing a judgement or opinion. We want to know what is the situation according to the best of your knowledge, even in absence of definitive information. |
| **Sexual contacts** | We use 'sexual contacts' as a broad term to refer to intercourse or other sexual acts. Their contents may not be the same in different populations. |
| **Sustainability** | In the context of behavioural and second generation surveillance systems, we understand 'sustainability' as the ability for the surveillance system to perform its functions over time. This is dependent upon the availability of appropriate resources, such as financing, expertise, political commitment, etc. |
| **WHO recommendations for "Second Generation Surveillance System" in low-level and concentrated epidemics** [Source: UNAIDS/WHO - Working Group on Global HIV/AIDS and STI Surveillance. Guidelines for second generation HIV surveillance. Geneva: UNAIDS/WHO; 2000] | **Recommendations for surveillance in a low-level epidemic**  • Cross-sectional surveys of behaviour in sub-populations with risk behaviour  • Surveillance of STIs and other biological markers of risk  • HIV surveillance in sub-populations at risk  • HIV and AIDS case reporting  • Tracking of HIV in donated blood  **Recommendations for surveillance in a concentrated epidemic**  HIV surveillance in a concentrated epidemic will contain all of the elements recommended for a low-level epidemic, but will add elements that focus more on the intersection between groups with different levels of risk.  • HIV and behavioural surveillance in sub-populations with risk behaviour  • HIV and behavioural surveillance in bridging groups  • Cross-sectional surveys of behaviour in the general population  • HIV sentinel surveillance in the general population, urban areas |
